# Paliperidone ER in the Treatment of Borderline Personality Disorder: A Pilot Study of Efficacy and Tolerability

**DOI:** 10.1155/2011/680194

**Published:** 2011-08-04

**Authors:** Silvio Bellino, Paola Bozzatello, Camilla Rinaldi, Filippo Bogetto

**Affiliations:** Centre for Personality Disorders, Unit of Psychiatry 1, Department of Neurosciences, University of Turin, via Cherasco 11, 10126 Turin, Italy

## Abstract

Antipsychotics are recommended for the treatment of impulsive dyscontrol and cognitive perceptual symptoms of borderline personality disorder (BPD). Three reports supported the efficacy of oral risperidone on BPD psychopathology. Paliperidone ER is the metabolite of risperidone with a similar mechanism of action, and its osmotic release reduces plasmatic fluctuations and antidopaminergic effects. The aim of this study is to evaluate efficacy and safety of paliperidone ER in BPD patients. 18 outpatients with a DSM-IV-TR diagnosis of BPD were treated for 12 weeks with paliperidone ER (3–6 mg/day). They were assessed at baseline, week 4, and week 12, using the CGI-Severity item, the BPRS, the HDRS, the HARS, the SOFAS, the BPD Severity Index (BPDSI), and the Barratt Impulsiveness Scale (BIS-11). Adverse events were evaluated with the DOTES. Paliperidone ER was shown to be effective and well tolerated in reducing severity of global symptomatology and specific BPD symptoms, such as impulsive dyscontrol, anger, and cognitive-perceptual disturbances. Results need to be replicated in controlled trials.

## 1. Introduction

Borderline personality disorder (BPD) is characterized by a pervasive pattern of instability in interpersonal relationships, self-image, and affects as well as impulsive dyscontrol that begins by early adulthood and appears in a variety of contexts [1].

Although psychotherapy plays a significant role in the treatment of BPD, focusing on maladaptive personality traits and interpersonal relationship patterns, pharmacotherapy is indicated by the treatment guidelines of the American Psychiatric Association [2, 3] to manage vulnerability traits, state symptoms, and acute relapses.

The availability of second-generation antipsychotics with a favourable tolerability profile has offered recent treatment options in the management of BPD patients. In particular, atypical antipsychotics are associated with fewer extrapyramidal adverse effects, a lower risk of tardive dyskinesia, and an improvement in cognitive functions [4–6].

Newer antipsychotics promise to be also more efficacious than traditional neuroleptics, probably due to their dual mechanism of action, targeting dysfunctions of both serotonergic and dopaminergic systems. These drugs produce their effects primarily on cognitive perceptual disturbances, such as transient paranoid or dissociative symptoms but also on symptoms of depression, anxiety, impulsivity, and aggressiveness.

Three reports supported the efficacy of oral risperidone on a large spectrum of BPD psychopathology, including affective instability, disturbed relationships, impulsivity, aggressiveness, hostility, and cognitive-perceptual impairment [7–9]. Paliperidone ER (9-hydroxyrisperidone) is a benzisoxazole derivative and the main active metabolite of risperidone. The efficacy and tolerability profile of this molecule is modulated both by drug-membrane interaction and drug-receptor action [10]. Paliperidone is an antagonist at dopamine receptor type 2 and serotonin receptor subtype 2A, with a greater affinity for 5HT2A receptor blockade relative to D2 receptor blockade. This drug has also some affinity for H1-histaminergic receptors and for *α*_1_- and *α*_2_-adrenergic receptors, whereas it lacks significant interactions with muscarinic receptors and with *β*1- and *β*2-adrenergic receptors [11–13]. Paliperidone does not undergo significant hepatic metabolism and is unlikely to induce drug-drug interactions [14–17]. Some authors suggested that paliperidone plays a predominant role in the increase of prolactinemia induced by risperidone [18, 19]. In fact, paliperidone was reported to rise prolactin to a higher serum level than risperidone [15, 20–22]. This issue is still debated, and other authors found that paliperidone and risperidone produced the same increase of prolactinemia over a period of 6 days [23].

Paliperidone extended release (ER) is compounded using osmotic-controlled dose-release system, a technology that reduces plasma level fluctuations of the immediate-release formulation [24–26]. This mechanism is expected to provide several advantages: a stable plasmatic level allows to avoid initial dosage adjustments and lowers the risk of antidopaminergic adverse effects. Another benefit is represented by once-daily administration, that simplifies treatment regimen and may improve patients' adherence.

Several clinical trials evaluated effectiveness of paliperidone ER in treating schizophrenia [15, 22, 27, 28]. In particular, recent studies ascertained significant improvements in positive symptoms, negative symptoms, depression and anxiety, uncontrolled hostility/excitement, and disorganized thoughts [27, 29]. At the moment, no data regarding the use of paliperidone ER in the treatment of personality disorders have been published.

The present paper is a pilot study with the aim of testing the efficacy and tolerability of paliperidone ER in patients with borderline personality disorder.

## 2. Materials and Methods

Consecutive outpatients aged between 18 and 60 years, who received a diagnosis of borderline personality disorder, were included in this study. Patients attended the Service for Personality Disorders, Unit of Psychiatry 1, Department of Neurosciences, University of Turin, Italy. Diagnosis of borderline personality disorder was made by an experienced clinician (S.B.) using DSM-IV-TR criteria [1] and performing the Structured Clinical Interview for DSM-IV Axis II Disorders [30].

Patients were required to fulfil exclusion criteria: a lifetime diagnosis of dementia, delirium, or other cognitive disorders; schizophrenia or other psychotic disorders; bipolar disorders; a co occurring major depressive episode; substance/alcohol abuse in the last six months; hyperprolactinemia at baseline.

Administration of psychotropic medications and/or psychotherapy in the three months before recruitment was excluded too. 

Female patients in childbearing age were excluded if they were not using adequate birth control methods (according to the judgment of the clinician). 

Each patient participated voluntarily in the study after providing a written informed consent. Declaration of Helsinki guidelines were followed, and approval by local Ethics Committee was obtained. 

Patients included in the study were treated for 12 weeks with open-label paliperidone ER 3–6 mg/day. No other psychotropic drug or psychological intervention was allowed during the trial.

Participants in the study were repeatedly assessed (at baseline, week 4, and week 12) using the following assessment instruments:

1. the Clinical Global Impression Scale-Severity item (CGI-S) [31];
2. the Brief Psychiatric Rating Scale (BPRS) [32];
3. the Hamilton Depression Rating Scale (HAM-D) [33];
4. the Hamilton Anxiety Rating Scale (HAM-A) [34];
5. the Social Occupational Functioning Assessment Scale (SOFAS) [35];
6. the Barratt Impulsiveness Scale-version 11 (BIS-11) [36];
7. the Borderline Personality Disorder Severity Index (BPDSI) [37].

The BPDSI is a semistructured clinical interview assessing frequency and severity of BPD-related symptoms. The interview consists of eight items scored on 10-point frequency scales (0 = never; 10 = daily), including “abandonment”, “interpersonal relationships”, “impulsivity”, “parasuicidal behavior”, “affective instability”, “emptiness”, “outbursts of anger”, “dissociation and paranoid ideation”, and one item scored on a 4-point severity scale, concerning “identity”. The BPDSI showed excellent reliability coefficients and good validity indices according to Arntz et al. [37].

The BIS-11 is a 30-item self-report questionnaire assessing the personality trait of impulsivity on a 4-point Likert Scale [38]. Higher scores for each item indicate higher levels of impulsivity. Twelve items are reversescored, in order to avoid response sets. The BIS-11 showed adequate reliability and construct validity in both US [39] and Italian [40] samples.

Assessment was performed by an investigator (P.B.) who was unaware of the dosing strategy. Prior to this study, the interviewer received training sessions on the BPDSI. 

Adverse effects were assessed using the Dosage Record and Treatment Emergent Symptoms Scale (DOTES) [41].

 Serum prolactin level was measured at baseline, after four and twelve weeks of treatment. Blood samples were collected in fasting patients two hours after they woke up. Hyperprolactinemia was defined as a level of serum prolactin ≥ 20 ng/mL in males and ≥ 25 ng/mL in females [42, 43]. Body weight was measured at baseline and endpoint. Weight gain at least 7% of baseline was considered significant [44]. 

Statistics were performed on the scores of each rating scale with the analysis of variance (ANOVA) for repeated measures with Bonferroni correction for multiple comparisons (software system SPSS, version 17.0, SPSS Inc., Chicago, Ill, 2008). *P* values were considered significant when ≤ 0.05.

## 3. Results

Initial sample was made of eighteen patients. They were five males and thirteen females, with a mean age ±SD = 24.3 ± 5.4. Fourteen patients (77.8%) completed the trial period. Four patients (1 male and 3 females, 22.2%) dropped out in the first four weeks of treatment: three patients due to noncompliance, one female patient for hyperprolactinemia. The fourteen patients who completed the trial had a mean age of 25.2 ± 4.9 years; they were four males and ten females. The mean ± SD daily dose of paliperidone ER was 4.8 ± 1.5 mg/day. 

Results of ANOVA with Bonferroni correction applied to rating scales scores are reported in Tables 1, 2, and 3. 

A statistically significant improvement was found for CGI severity item (*P* = 0.001), BPRS mean score (*P* = 0.001), SOFAS mean score (*P* = 0.001), BIS-11 mean score (*P* = 0.005), BPDSI total score (*P* = 0.001), and items “impulsivity” (*P* = 0.001), “outbursts of anger” (*P* = 0.01), and “dissociative symptoms/paranoid ideation” (*P* = 0.002). On the contrary, there were no significant changes of HAM-D and HAM-A mean score and BPDSI items “abandonment,” “interpersonal relationships,” “identity,” “parasuicidal behaviour,” “affective instability,” and “emptiness.”

As far as paliperidone tolerability is concerned, only one case of severe side effect (hyperprolactinemia) inducing treatment discontinuation was observed in the initial group of eighteen subjects (5.6%). Adverse effects recorded in the final sample of fourteen patients were mild to moderate and included insomnia (*n* = 3, 21.4%), gastrointestinal symptoms (*n* = 2, 14.3%), agitation (*n* =1, 7.1%), and extrapyramidal symptoms (tremor) (*n* = 1, 7.1%). Six patients (42.9%) had at least one adverse effect during treatment period. No cases of significant weight gain (≥7% of baseline) were recorded. Mean weight gain ± SD was 0.7 ± 0.8 kg.

## 4. Discussion

Findings of this pilot study of paliperidone ER in patients with borderline personality disorder showed significant clinical and functional improvements, as measured with CGI severity item, BPRS, SOFAS, Barratt Impulsiveness Scale, BPDSI total score, and items “impulsivity,” “outbursts of anger,” and “dissociative symptoms/paranoid ideation”.

Because of the lack of trials of paliperidone in the treatment of BPD, these results can only be compared with data concerning the effects of other second-generation antipsychotics in this personality disorder. A particular interest is raised by clinical trials of risperidone, a drug with a chemical structure very similar to paliperidone (actually paliperidone is the active metabolite of risperidone). 

The significant changes of the rating scale scores after treatment, in particular the reduction of the CGI-S, BPRS, and total BPDSI scores and the increase of the SOFAS score, indicated that paliperidone ER was efficacious on a large spectrum of BPD psychopathology. The improvement of global symptomatology and social functioning was reported by previous open and controlled studies concerning BPD treatment with atypical antipsychotics, such as risperidone [8, 45], clozapine [46, 47], olanzapine [48, 49], quetiapine [50–56], and aripiprazole [57–60].

In addition to the improvement of global psychopathology, paliperidone ER produced in our patients significant effects on specific symptom dimensions, such as dyscontrol of impulsivity and cognitive perceptual distortions. Measures with the clinician-rated BPDSI and the self-report BIS-11 were concordant and indicated that paliperidone was effective on impulsive-behavioral dyscontrol symptoms and outbursts of anger. Efficacy of second generation antipsychotics on this cluster of symptoms was already reported by several trials of olanzapine [48, 61, 62] and quetiapine [50–53, 55, 56]. Data concerning the effect on impulsivity of other antipsychotics are less consistent. This effect was never reported in trials of clozapine, and it was found in two pilot studies of risperidone [9, 45] and in one pilot study of aripiprazole in treatment refractory BPD patients [60]. Controlled trials of these two drugs did not list improvement of impulsive behaviors among their findings [7, 58]. Discordant data on this issue, particularly concerning risperidone, suggest the need to replicate our initial data on paliperidone, before drawing any conclusion for clinical practice.

Measures with the BPDSI item “dissociation and paranoid ideation” in our sample provided initial evidence that paliperidone ER was effective in treating cognitive and perceptual disturbances, such as ideas of reference, illusions, and dissociative symptoms. The efficacy of second-generation antipsychotics on cognitive and perceptual symptoms was considered in rather few studies of BPD patients. It was reported in several trials with clozapine [46, 47, 63], in two studies with risperidone [7, 9], one with olanzapine [64], one with ziprasidone [65], and one with aripiprazole [60]. Further investigations are needed to confirm this antipsychotic effect in BPD.

Concerning tolerability, adverse effects in the final sample of BPD patients were insomnia, gastrointestinal disturbances, agitation, and extrapyramidal symptoms. Side effects were mild to moderate in severity. Only one case of hyperprolactinemia induced treatment discontinuation. It is noticeable that we found a low incidence of hyperprolactinemia, compared with previous reports in patients with schizophrenia [20–22, 66]. No cases of significant weight gain were recorded, in contrast with a recent estimate of 8% in patients with schizophrenia or bipolar disorder [44]. A possible explanation for these differences is that we used low to moderate doses of paliperidone (3–6 mg/day). Usually, doses of paliperidone in patients with schizophrenia were higher, and some authors retained that hyperprolactinemia induced by paliperidone was dose related [21, 67]. The pattern of side effects in our sample was generally concordant with investigations of paliperidone in the treatment of schizophrenia [15, 27, 29, 67–69].

## 5. Conclusions

Initial findings were promising and indicated that low to moderate doses of the new antipsychotic paliperidone ER were efficacious in reducing severity of global symptomatology and social impairment and in treating two specific clusters of BPD symptoms, impulsive behavioral dyscontrol, and cognitive-perceptual disturbance. The level of tolerability recorded in our patients was favourable, with low incidence of hyperprolactinemia. 

Limitations of the present study were the small sample size and the lack of a control group. However, this paper was aimed to provide initial data on the use of paliperidone ER in BPD treatment and promote larger trials with a double-blind controlled design.

## Figures and Tables

**Table 1:** Results of analysis of variance (ANOVA) for repeated measures with Bonferroni correction for multiple comparisons, performed with rating scales for BPD-related symptoms and social functioning in patients treated with paliperidone ER (*N* = 14).

Variable		Mean	SD	SE	*P*
CGI severity item score	T0	4.50	0.51	0.12	0.001
	T1	4.28	0.46	0.11	
	T2	3.72	0.46	0.11	
BPRS score	T0	42.28	4.92	1.16	0.001
	T1	38.28	4.61	1.09	
	T2	35.11	3.86	0.91	
HARS score	T0	18.17	4.20	0.99	0.262
	T1	13.83	4.63	1.09	
	T2	15.50	4.90	1.16	
HDRS score	T0	10.50	3.88	0.92	0.120
	T1	8.67	2.61	0.62	
	T2	8.39	2.28	0.54	
SOFAS score	T0	48.78	6.26	1.47	0.001
	T1	53.06	5.67	1.34	
	T2	59.33	6.28	1.48	

**Table 2:** Results of analysis of variance (ANOVA) for repeated measures with Bonferroni correction for multiple comparisons, performed with BPDSI total score and items “abandonment”, “interpersonal relationship”, “identity”, and “impulsivity”.

Variable		Mean	SD	SE	*P*
BPDSI total score	T0	51.94	3.94	1.478	0.001
	T1	47.61	4.40	1.632	
	T2	44.45	3.78	1.423	
Abandonment	T0	6.65	1.51	0.36	1.000
	T1	6.35	1.41	0.33	
	T2	6.37	1.58	0.37	
Interpersonal relationships	T0	7.18	1.36	0.32	0.750
	T1	6.72	1.25	0.30	
	T2	6.69	1.11	0.26	
Identity	T0	2.81	0.75	0.18	1.000
	T1	2.79	0.73	0.17	
	T2	2.83	0.70	0.17	
Impulsivity	T0	7.28	0.77	0.18	0.001
	T1	6.17	0.89	0.21	
	T2	5.69	1.14	0.27	

**Table 3:** Results of analysis of variance (ANOVA) for repeated measures with Bonferroni correction for multiple comparisons, performed with BPDSI items “parasuicidal behaviour,” “affective instability,” “emptiness,” “outbursts of anger,” and “dissociation and paranoid ideation” and with BIS-11 score.

Variable		Mean	SD	SE	*P*
Parasuicidal behaviour	T0	3.46	2.05	0.48	0.101
	T1	2.78	2.03	0.48	
	T2	2.08	1.53	0.36	
Affective instability	T0	5.19	1.26	0.30	0.083
	T1	4.54	0.87	0.20	
	T2	4.42	0.86	0.20	
Emptiness	T0	6.41	1.73	0.41	1.000
	T1	6.22	1.62	0.38	
	T2	6.45	1.43	0.34	
Outbursts of anger	T0	7.38	0.60	0.14	0.01
	T1	6.77	0.72	0.17	
	T2	6.38	1.05	0.25	
Dissociation and paranoid ideation	T0	6.36	1.14	0.27	0.002
	T1	5.98	1.04	0.25	
	T2	4.84	1.18	0.28	
BIS score	T0	71.17	7.79	1.83	0.005
	T1	65.17	6.31	1.49	
	T2	62.28	6.43	1.52	

## References

[1] American Psychiatric Association (2000). *Diagnostic and Statistical Manual of Mental Disorders*.

[2] American Psychiatric Association (2001). *Practice Guidelines for the Treatment of Patients with Borderline Personality Disorder*.

[3] Oldham JM (2005). *Guideline Watch: Practice Guideline for the Treatment of Patients with Borderline Personality Disorder*.

[4] Meltzer HY, McGurk SR (1999). The effects of clozapine, risperidone, and olanzapine on cognitive function in schizophrenia. *Schizophrenia Bulletin*.

[5] Correll CU, Leucht S, Kane JM (2004). Lower risk for tardive dyskinesia associated with second-generation antipsychotics: a systematic review of 1-year studies. *American Journal of Psychiatry*.

[6] Van Den Eynde F, De Saedeleer S, Naudts K (2009). Quetiapine treatment and improved cognitive functioning in borderline personality disorder. *Human Psychopharmacology*.

[7] Szigethy EM, Schulz SC (1997). Risperidone in comorbid borderline personality disorder and dysthymia. *Journal of Clinical Psychopharmacology*.

[8] Rocca P, Marchiaro L, Cocuzza E, Bogetto F (2002). Treatment of borderline personality disorder with risperidone. *Journal of Clinical Psychiatry*.

[9] Friedel RO, Jackson WT, Huston CS, May RS, Kirby NL, Stoves A (2008). Risperidone treatment of borderline personality disorder assessed by a borderline personality disorder-specific outcome measure: a pilot study. *Journal of Clinical Psychopharmacology*.

[10] Jutila A, Söderlund T, Pakkanen AL, Huttunen M, Kinnunen PKJ (2001). Comparison of the effects of clozapine, chlorpromazine, and haloperidol on membrane lateral heterogeneity. *Chemistry and Physics of Lipids*.

[11] Megens AAHP, Awouters FHL, Schotte A (1994). Survey on the pharmacodynamics of the new antipsychotic risperidone. *Psychopharmacology*.

[12] Schotte A, Janssen PFM, Gommeren W (1996). Risperidone compared with new and reference antipsychotic drugs: in vitro and in vivo receptor binding. *Psychopharmacology*.

[13] Leysen JE, Ellenbroek BA, Cools AR (2000). Receptor profile of antipsychotics. *Atypical Antipsychotics*.

[14] Vermeir M, Boom S, Naessens I, Talluri K, Eerdekens M (2005). Absorbtion, metabolism and excretion of a single oral dose of 14C-paliperidone 1mg in healthy subjects. *European Neuropsychopharmacology*.

[15] Marder SR, Kramer M, Ford L (2007). Efficacy and safety of paliperidone extended-release tablets: results of a 6-week, randomized, placebo-controlled study. *Biological Psychiatry*.

[16] Lautenschlager M, Heinz A (2008). Paliperidone-ER: first atypical antipsychotic with oral extended release formulation. *Expert Review of Neurotherapeutics*.

[17] Nasrallah HA (2008). Atypical antipsychotic-induced metabolic side effects: insights from receptor-binding profiles. *Molecular Psychiatry*.

[18] Knegtering R, Baselmans P, Castelein S, Bosker F, Bruggeman R, Van Den Bosch RJ (2005). Predominant role of the 9-hydroxy metabolite of risperidone in elevating blood prolactin levels. *American Journal of Psychiatry*.

[19] Melkersson KI (2006). Prolactin elevation of the antipsychotic risperidone is predominantly related to its 9-hydroxy metabolite. *Human Psychopharmacology*.

[20] Kane J, Canas F, Kramer M (2007). Treatment of schizophrenia with paliperidone extended-release tablets: a 6-week placebo-controlled trial. *Schizophrenia Research*.

[21] Davidson M, Emsley R, Kramer M (2007). Efficacy, safety and early response of paliperidone extended-release tablets (paliperidone ER): results of a 6-week, randomized, placebo-controlled study. *Schizophrenia Research*.

[22] Dolder C, Nelson M, Deyo Z (2008). Paliperidone for schizophrenia. *American Journal of Health-System Pharmacy*.

[23] Berwaerts J, Cleton A, Rossenu S (2010). A comparison of serum prolactin concentrations after administration of paliperidone extended-release and risperidone tablets in patients with schizophrenia. *Journal of Psychopharmacology*.

[24] Karlsson P, Dencker E, Nyberg S (2005). Pharmacokinetic and dopamine D2 and serotonin 5-HT2A receptor occupancy of paliperidone in healthy subjects. *European Neuropsychopharmacology*.

[25] Karlsson P, Dencker E, Nyberg S (2006). Pharmacokinetics, dopamine D2 and serotonin 5-HT2A receptor occupancy and safety profile of paliperidone extended-release in healthy subjects. *Schizophrenia Research*.

[26] Conley R, Gupta SK, Sathyan G (2006). Clinical spectrum of the osmotic-controlled release oral delivery system (OROS), an advanced oral delivery form. *Current Medical Research and Opinion*.

[27] Canuso CM, Bossie CA, Turkoz I, Alphs L (2009). Paliperidone extended-release for schizophrenia: effects on symptoms and functioning in acutely ill patients with negative symptoms. *Schizophrenia Research*.

[28] Pani L, Marchese G (2009). Expected clinical benefits of paliperidone extended-release formulation when compared with risperidone immediate-release. *Expert Opinion on Drug Delivery*.

[29] Meltzer HY, Bobo WV, Nuamah IF (2008). Efficacy and tolerability of oral paliperidone extended-release tablets in the treatment of acute schizophrenia: pooled data from three 6-week, placebo-controlled studies. *Journal of Clinical Psychiatry*.

[30] First MB, Gibbon M, Spitzer RL, Williams JBW, Benjamin LS (1997). *Structured Clinical Interview for DSM-IV disorders Axis II (SCID-II)*.

[31] Guy W, US Dept Health, Education, and Welfare publication (ADM) (1976). Clinical global impression (C.G.I.). *ECDEU Assessment Manual for Psychopharmacology*.

[32] Ventura J, Green M, Shaner A, Liberman R (1993). Training and quality assurance with the Brief Psychiatry Rating Scale: “the drift busters”. *International Journal of Psychiatric Research*.

[33] Hamilton M (1960). A rating scale for depression. *Journal of Neurology, Neurosurgery, and Psychiatry*.

[34] Hamilton M (1959). The assessment of anxiety states by rating. *The British Journal of Medical Psychology*.

[35] Goldman HH, Skodol AE, Lave TR (1992). Revising axis V for DSM-IV: a review of measures of social functioning. *American Journal of Psychiatry*.

[36] Barratt ES (1965). Factor analysis of some psychometric measures of impulsiveness and anxiety. *Psychological Reports*.

[37] Arntz A, Van den Hoorn M, Cornelis J, Verheul R, Van den Bosch WMC, De Bie AJHT (2003). Reliability and validity of the borderline personality disorder severity index. *Journal of Personality Disorders*.

[38] Barratt ES, Monahan J, Steadman HJ (1994). Impulsiveness and aggression. *Violence and Mental Disorder: Developments in Risk Assessment*.

[39] Patton JH, Stanford MS, Barratt ES (1994). Factor structure of the Barratt Impulsiveness Scale. *Journal of Clinical Psychology*.

[40] Fossati A, Di Ceglie A, Acquarini E, Barratt ES (2001). Psychometric properties of an Italian version of the Barrat Impulsiveness Scale-11 (BIS-11) in nonclinical subjects. *Journal of Clinical Psychology*.

[41] Guy W, US Department of Health, Education, and Welfare, Public Health Service, Alcohol, Drug Abuse, and Mental Health Administration, NIMH Psychopharmacology Research Branch, Division of Extramural Research Programs (1976). Dosage Record and Treatment Emergent Symptoms scale (DOTES). *ECDEU Assessment Manual for Psychopharmacology—Revised*.

[42] Haddad PM, Wieck A (2004). Antipsychotic-induced hyperprolactinaemia: mechanisms, clinical features and management. *Drugs*.

[43] Mancini T, Casanueva FF, Giustina A (2008). Hyperprolactinemia and prolactinomas. *Endocrinology and Metabolism Clinics of North America*.

[44] Harrington CA, English C (2010). Tolerability of paliperidone: a meta-analysis of randomized, controlled trials. *International Clinical Psychopharmacology*.

[45] Díaz-Marsá M, Galian M, Montes A (2008). Long-acting injectable risperidone in treatment resistant borderline personality disorder. A small series report. *Actas Espanolas de Psiquiatria*.

[46] Frankerburg FR, Zanarini MC (1993). Clozapine treatment of borderline patients: a preliminary study. *Comprehensive Psychiatry*.

[47] Parker GF (2002). Clozapine and borderline personality disorder. *Psychiatric Services*.

[48] Schulz SC, Camlin KL, Berry SA, Jesberger JA (1999). Olanzapine safety and efficacy in patients with borderline personality disorder and comorbid dysthymia. *Biological Psychiatry*.

[49] Bogenschutz MP, Nurnberg HG (2004). Olanzapine versus placebo in the treatment of borderline personality disorder. *Journal of Clinical Psychiatry*.

[50] Adityanjee A, Schulz SC (2002). Clinical use of quetiapine in disease states other than schizophrenia. *Journal of Clinical Psychiatry*.

[51] Hilger E, Barnas C, Kasper S (2003). Quetiapine in the treatment of borderline personality disorder. *The World Journal of Biological Psychiatry*.

[52] Villeneuve E, Lemelin S (2005). Open-label study of atypical neuroleptic quetiapine for treatment of borderline personality disorder: impulsivity as main target. *Journal of Clinical Psychiatry*.

[53] Bellino S, Paradiso E, Bogetto F (2006). Efficacy and tolerability of quetiapine in the treatment of borderline personality disorder: a pilot study. *Journal of Clinical Psychiatry*.

[54] Perrella C, Carrus D, Costa E, Schifano F (2007). Quetiapine for the treatment of borderline personality disorder; an open-label study. *Progress in Neuro-Psychopharmacology and Biological Psychiatry*.

[55] Adityanjee A, Romine A, Brown E, Thuras P, Lee S, Schulz SC (2008). Quetiapine in patients with borderline personality disorder: an open-label trial. *Annals of Clinical Psychiatry*.

[56] Van Den Eynde F, Senturk V, Naudts K (2008). Efficacy of quetiapine for impulsivity and affective symptoms in borderline personality disorder. *Journal of Clinical Psychopharmacology*.

[57] Mobascher A, Mobascher J, Schlemper V, Winterer G, Malevani J (2006). Aripiprazole pharmacotherapy of borderline personality disorder: a series of three consecutive case reports. *Pharmacopsychiatry*.

[58] Nickel MK, Muehlbacher M, Nickel C (2006). Aripiprazole in the treatment of patients with borderline personality disorder: a double-blind, placebo-controlled study. *American Journal of Psychiatry*.

[59] Nickel MK (2007). Aripiprazole treatment of patients with borderline personality disorder. *Journal of Clinical Psychiatry*.

[60] Bellino S, Paradiso E, Bogetto F (2008). Efficacy and tolerability of aripiprazole augmentation in sertraline-resistant patients with borderline personality disorder. *Psychiatry Research*.

[61] Zanarini MC, Frankenburg FR, Parachini EA (2004). A preliminary, randomized trial of fluoxetine, olanzapine, and the olanzapine-fluoxetine combination in women with borderline personality disorder. *Journal of Clinical Psychiatry*.

[62] Soler J, Pascual JC, Campins J (2005). Double-blind, placebo-controlled study of dialectical behavior therapy plus olanzapine for borderline personality disorder. *American Journal of Psychiatry*.

[63] Benedetti F, Sforzini L, Colombo C, Maffei C, Smeraldi E (1998). Low-dose clozapine in acute and continuation treatment of severe borderline personality disorder. *Journal of Clinical Psychiatry*.

[64] Zanarini MC, Frankenburg FR (2001). Olanzapine treatment of female borderline personality disorder patients: a double-blind, placebo-controlled pilot study. *Journal of Clinical Psychiatry*.

[65] Pascual JC, Oller S, Soler J, Barrachina J, Alvarez E, Pérez V (2004). Ziprasidone in the acute treatment of borderline personality disorder in psychiatric emergency services. *Journal of Clinical Psychiatry*.

[66] Bostwick JR, Guthrie SK, Ellingrod VL (2009). Antipsychotic-induced hyperprolactinemia. *Pharmacotherapy*.

[67] Spina E, Cavallaro R (2007). The pharmacology and safety of paliperidone extended-release in the treatment of schizophrenia. *Expert Opinion on Drug Safety*.

[68] Marino J, Caballero J (2008). Paliperidone extended-release for the treatment of schizophrenia. *Pharmacotherapy*.

[69] Tzimos A, Samokhvalov V, Kramer M (2008). Safety and tolerability of oral paliperidone extended-release tablets in elderly patients with schizophrenia: a double-blind, placebo-controlled study with six-month open-label extension. *American Journal of Geriatric Psychiatry*.

